# Melanoma stem cells promote metastasis via exosomal miR-1268a inactivation of autophagy

**DOI:** 10.1186/s40659-022-00397-z

**Published:** 2022-10-01

**Authors:** Xiaoshuang Li, Doudou Liu, Hao Chen, Bin Zeng, Qiting Zhao, Yuhan Zhang, Yuting Chen, Jianyu Wang, H. Rosie Xing

**Affiliations:** 1 Institute of Life Sciences, Chongqing Medical University, Chongqing, China; 2 State Key Laboratory of Ultrasound in Medicine and Engineering, College of Biomedical Engineering, Chongqing Medical University, Chongqing, 400016 China; 3 Chongqing Key Laboratory of Biomedical Engineering, Chongqing Medical University, Chongqing, 400016 China

**Keywords:** Melanoma stem cells, Exosomes, miR-1268a, Metastasis, Autophagy

## Abstract

**Background:**

Metastatic melanoma has a high mortality rate and poor survival. This is associated with efficient metastatic colonization, but the underlying mechanisms remain elusive. Communication between cancer stem cells (CSCs) and cancer cells plays an important role in metastatic dissemination. Whether cancer stem cells can alter the metastatic properties of non-CSC cells; and whether exosomal crosstalk can mediate such interaction, have not been demonstrated in melanoma prior to this report.

**Results:**

The results revealed that exosomes secreted by highly metastatic melanoma CSCs (OL-SCs) promoted the invasiveness of the low metastatic melanoma cells (OL) and accelerated metastatic progression. miR-1268a was up-regulated in cells and exosomes of OL-SCs. Moreover, OL-SCs-derived exosomal miR-1268a, upon taking up by OL cells, promoted the metastatic colonization ability of OL cells in vitro and in vivo. In addition, the pro-metastatic activity of exosomal miR-1268a is achieved through inhibition of autophagy.

**Conclusion:**

Our study demonstrates that OL cells can acquire the “metastatic ability” from OL-SCs cells. OL-SCs cells achieves this goal by utilizing its exosomes to deliver functional miRNAs, such as miR-1268a, to the targeted OL cells which in turn augments metastatic colonization by inactivating the autophagy pathway in OL cells.

**Supplementary Information:**

The online version contains supplementary material available at 10.1186/s40659-022-00397-z.

## Introduction

Melanoma is an aggressive cancer with high mortality. The mean survival of metastatic melanoma is less than 1 year [1–4]. Although the new line of therapies (targeted therapy and immunotherapies) has brought new options and prolonged survival to metastatic melanoma patients, development of resistance remains the bottleneck for melanoma management. Efficient metastatic colonization at the involved organ is a feature of metastatic melanoma. However, the underlying mechanisms remain to be fully elucidated.

Tumor microenvironment (TME) plays a key role in tumor metastasis. In addition to cancer cells, TME also includes various cell types, extracellular matrix (ECM), growth factors, proteolytic enzymes and their inhibitors, as well as signaling molecules [5, 6]. Tumor progression is the outcome of the coordinated activities and interactions of these components. While cell-cell interactions in TME have been well appreciated, most attention has been paid to the interactions between cancer cells and a variety of non-cancerous cells (fibroblasts, vascular endothelial cells and immune cells) [7]. Less is known about the interactions among heterogeneous cancer cells, such as between CSCs and non-CSCs and the impact of such interactions on the course of cancer progression. Exosomes emerge as a new class of mediators for cell-cell interactions. Exosomes are membranous micro-vesicles with a diameter of 40–160 nm, which can be produced by different types of cells [8–10]. Exosomes play an important role in cell-to-cell communication by transferring biologically active substances (proteins, lipids, and non-coding RNAs) from donor cells to the recipient cells. Various studies have shown that exosomes are key mediators of cell-to-cell communication between tumor cells and stromal cells in both local and distant microenvironments [11]. Exosomes may modulate the biological functions, or cellular fate or properties of the target cells depending on the content of exosomes, and hence may influence the course of cancer initiation, progression or development of resistance to therapies [12–15].

The research on exosomes in cancer progression can be divided into three main categories [16, 17]: First, most of the studies have been focused on cancer cell-derived exosomes in modulating the functions of stromal cells [13, 18–21]. Second, there are increasing evidence demonstrating the ability of stromal cell in TME derived exosomes in modifying the behavior of cancer cells [22–24]. Third, few studies have shown exosomal crosstalk between cancer cells that promotes tumor metastasis or epithelial-mesenchymal transition [7, 25, 26]. In melanoma research, exosomes secreted by melanoma cells can cause normal melanocytes to undergo malignant transformation [27, 28], and can target surrounding stromal cells [29] such as mesenchymal stem cells [30, 31], immune cells [32], fibroblasts [33] to make a favorable microenvironment for melanoma growth and progression. These findings show that exosomes may play an important role in melanoma oncogenesis and progression. Whether the extravasated CSCs can alter the metastatic properties of neighboring non-CSCs through exosomal crosstalk, a new mechanism of metastatic colonization, has been rarely investigated by studies prior to this report.

In this study, a paired M14 melanoma derivative cell line, i.e., M14-OL and its CSC derivative cell line M14-OL-SCs, that we established and characterized were employed. We show that exosomal crosstalk between CSCs and non-CSCs is a new mechanism that underlies melanoma metastasis. Low metastatic melanoma cells (OL) can acquire the “metastatic ability” from highly metastatic melanoma CSCs (OL-SCs). OL-SCs achieve this goal by utilizing their exosomes to deliver functional miRNAs, such as miR-1268a, to the targeted OL cells which in turn augments metastatic colonization by inactivating the autophagy pathway in OL cells.

## Results

### Characterization of exosomes derived from OL and OL-SCs

**Fig.1:**
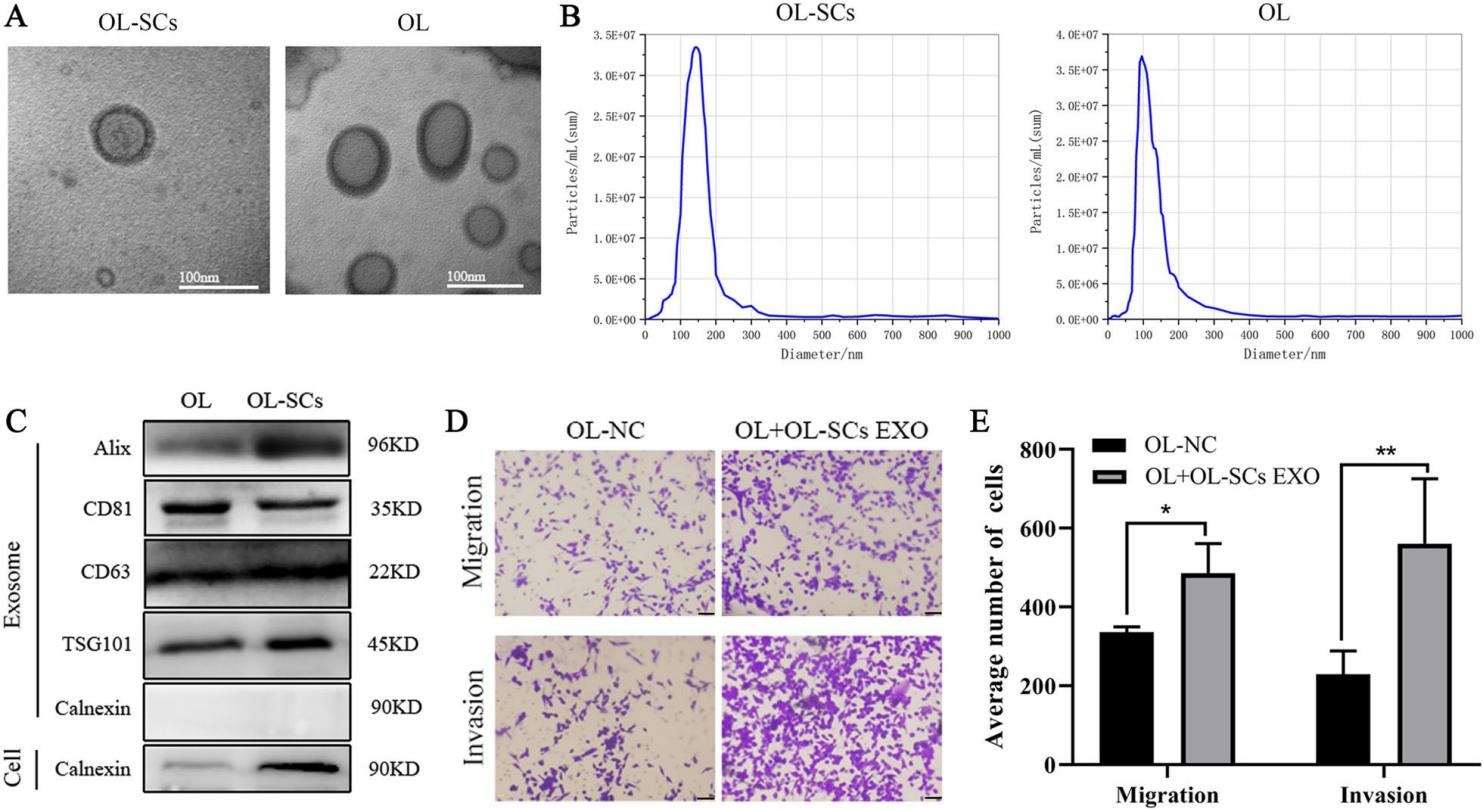
Exosome secreted by melanoma stem cells promote metastasis of melanoma. **A** The morphology of the obtained exosomes (OL-SCs-EXO、OL-EXO) was analyzed by Transmission electron microscopy (TEM) Scale bar, 100 nm. **B** Size distribution and concentration of exosomes derived from OL cells and OL-SCs cells was performed by Nanoparticle Tracking Analysis (NTA). **C** The expression of typical exosome marker proteins(Alix, CD63, CD81, TSG101, Calnexin) was detected by Western blot. **D**, **E** OL-SCs-EXO (40 μg) promoted migration and invasion ability of OL cells as revealed by Transwell assay. (Three replicate wells were set in each group, which were repeated 3 times.10 random fields from three replicate Transwell were counted. n = 3) Scale bar, 64 μm. Data were expressed as the mean $\pm$ SD, *p < 0.05, **p < 0.01, ***p < *0.001*

In the present study, we used a paired cell line OL and OL-SCs cells derived from M14 cells, which we have generated and characterized in previous studies. OL cells are spindle-shaped, grow adherently, exhibit an oligometastatic phenotype in vivo following tail vein injection in mice, and form a limited number of metastatic foci on mouse lungs (refer as oligometastatic) [34]. Melanoma Stem Cells (OL-SCs) were isolated and purified from OL cells, spherical in shape, grown in suspension, and expressed Aldh1, CD133, Nanog, Oct4 and Sox2 stemness markers. When OL-SCs were subcutaneously injected in nude mice, tumors of uniform size could be successfully formed after 16 days [35]. Exosomes were isolated and purified from the culture supernatants of OL and OL-SCs cells by ultracentrifugation (Sect. “Materials and methods”). The morphology of the obtained exosomes was analyzed by Transmission electron microscopy (TEM) for visualization of their round or elliptical membranous vesicle morphology (Fig. 1A); by Nanoparticle Tracking Analysis (NTA) for their concentration and size range (40 ~ 150 nm, with an average around 100 nm, Fig. 1B), and by Western blot for the expression of exosomal marker proteins Alix, CD63, CD81, TSG101 and exosome-negative marker protein Calnexin (Fig. 1C).The above results confirmed that the isolated vesicles were exosomes.

### Exosomes secreted by OL-SCs cells can promote the migration and invasion of the low-metastatic OL cells in vitro

At the involved organ, whether CSCs can enhance the metastasis of non-CSCs cancer cells that have low metastatic capability has not been characterized. We investigated the effects of OL-SCs-EXO on the metastatic function of OL cells in vitro. OL cells incubated with 40 μg OL-SCs-EXO displayed increased migration and invasion, measured by Transwell assays (Fig. 1D, E) compared to the control cells incubated with PBS (OL-NC).

**Fig.2:**
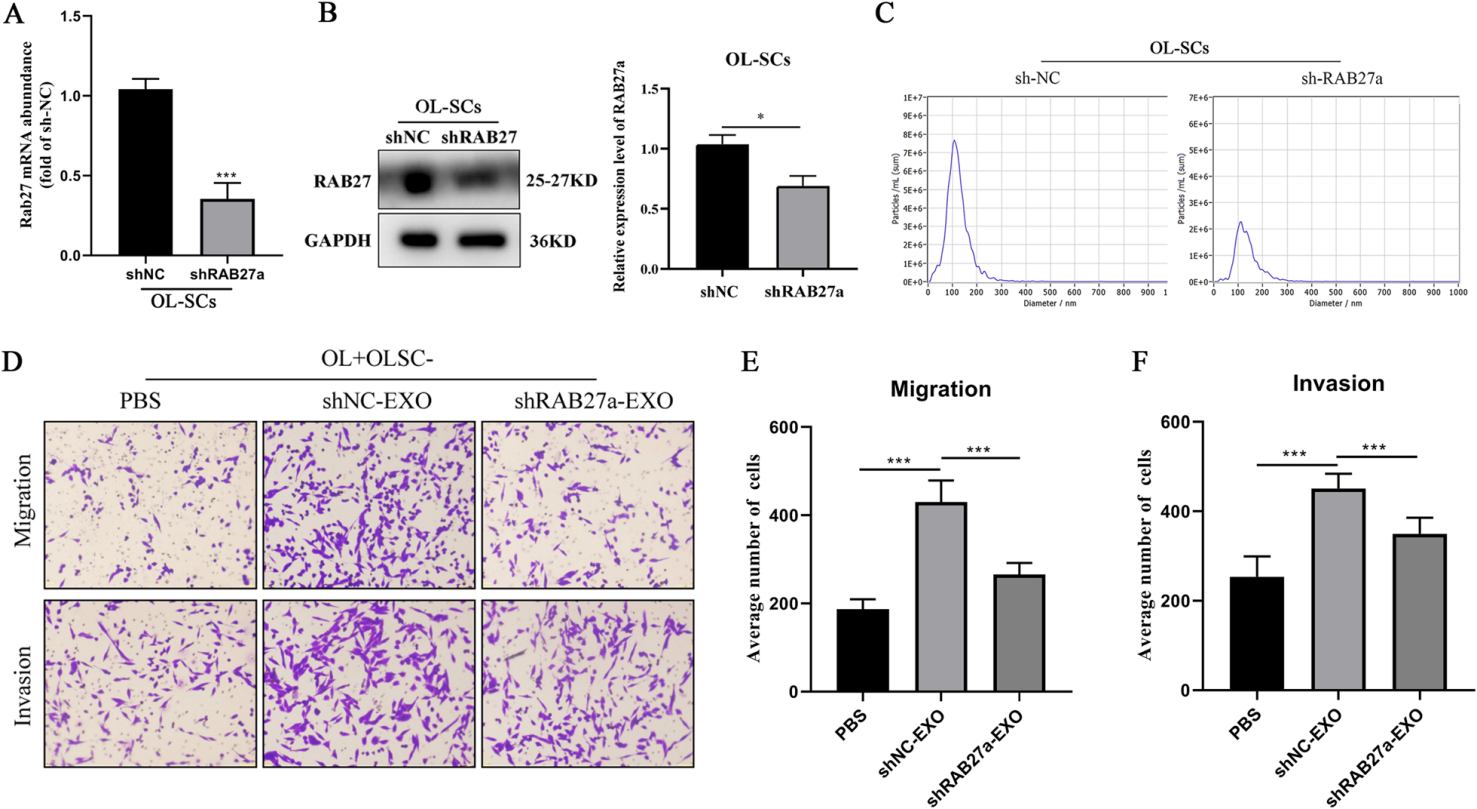
Reduction of OL-SCs exosomes secretion largely prevented the stimulatory effect of OL-SCs exosomes on OL cells migration and invasion in vitro. Exosome secretion was reduced by knocking down the key gene RAB27a that regulates the secretion and release of exosomes in OL-SCs cells. **A**, **B** RT-qPCR and Western blots were performed confirm effective silencing of RAB27a expression in OL-SCs cells. The right side of Figure B is the grayscale analysis statistical chart of WB (the ordinate is the target protein/GAPDH) **C** The concentration and size distribution of OL-SCs exosomes after RAB27a knocking down were confirmed by NTA. **D** The exosomes secreted by OL-SCs cells silenced by RAB27a gene inhibited the migration and invasion of OL cells by Transwell assay. **E**, **F** Quantitative analysis of migrating and invasive cells. (Three replicate wells were set in each group, which were repeated 3 times.10 random fields from three replicate Transwell were counted. n = 3) Scale bar, 64 μm. Data were expressed as the mean $\pm$ SD, *p < 0.05, **p < 0.01, ***p < *0.001*

Rab-GTPases play central roles in regulating multiple steps of vesicular trafficking. Importantly, the Rab-GTPases (RAB27a, RAB27b, RAB35, RAB11and RAB7) are shown to regulate multivesicular body (MVB) trafficking for exosomes release. Among them, RAB27, as a regulator of late endosomal docking with the plasma membrane, has been shown to be involved in exosome secretion [36, 37]. Knockdown of RAB27a can inhibit the secretion of exosomes by melanoma cancer cells [38]. To confirm that the effects we observed with the addition of OL-SCs-EXO were caused by the OL-SCs secreted exosomes, exosome secretion function of OL-SCs cells was reduced by RAB27a siRNA silencing (sh-RAB27a) (Sect. “Materials and methods”). Effective inhibition of RAB27a expression was confirmed by RT-qPCR (Fig. 2A) and WB (Fig. 2B), respectively. From the results of NTA, we can see that when the expression of RAB27a was silenced in OL-SCs cells, the concentration of exosomes decreased by about 3 times compared with the sh-NC group (Fig. 2C). As expected, inhibition of RAB27a resulted in reduced secretion of exosomes, largely prevented the increase of the number of migratory and invaded OL cells seen after co-culturing with OL-SCs-EXO (Fig. 2D, F).

Collectively, these observations demonstrate that OL-SCs-EXO can augment the metastatic function of OL cells in vitro. This led us to hypothesize that “*biologically active factors are transferred from OL-SCs to OL cells through OL-SCs-EXO which in turn enhances the metastatic ability of OL cells*”.

### OL-SCs secreted exosomal miR-1268a promotes the metastatic colonization ability of OL cells in vitro and in vivo

**Fig.3:**
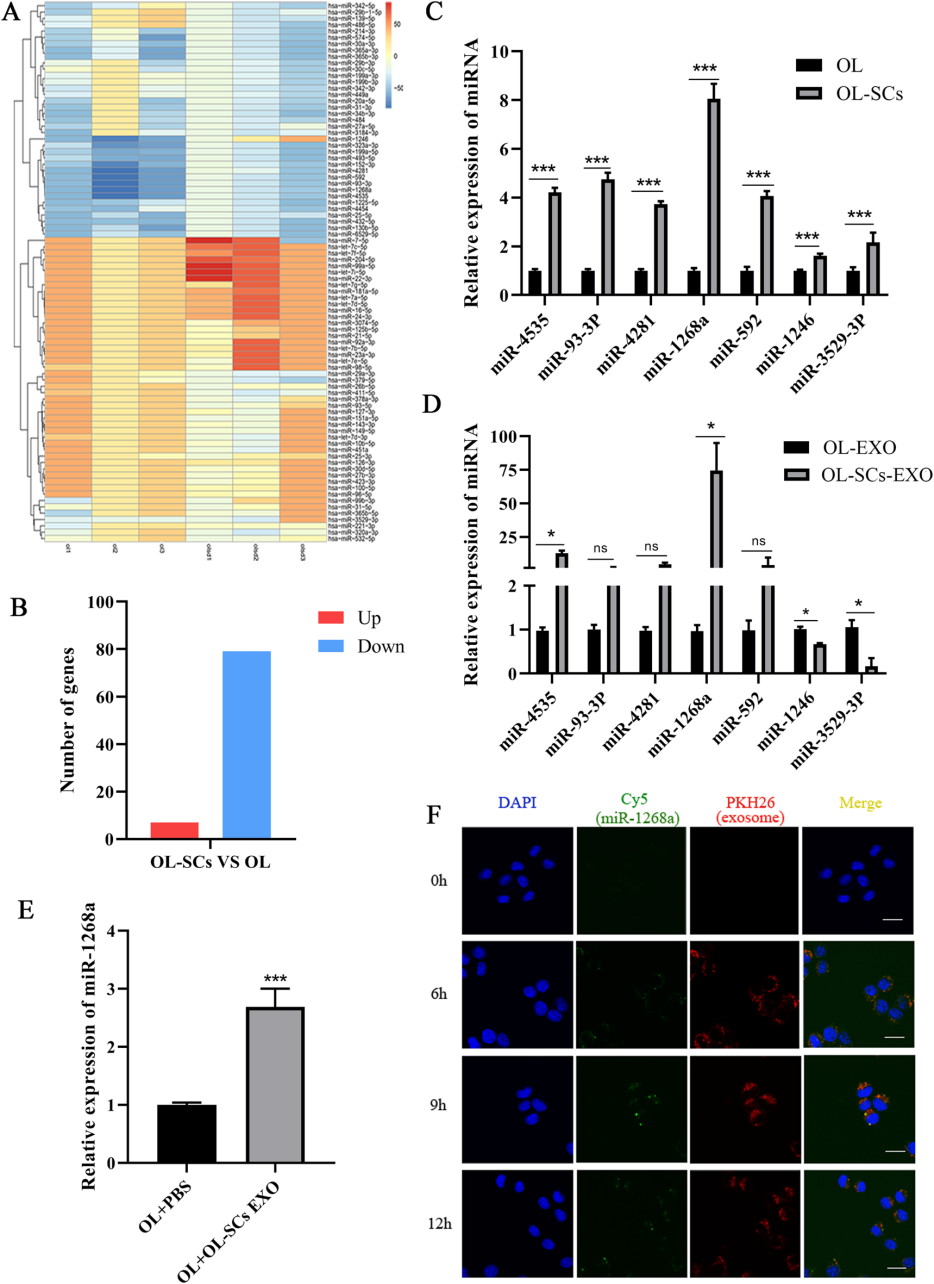
Cellular and exosomal miR-1268a were upregulated in melanoma stem cells and transferred to melanoma cells. **A**, **B** The heat map and histogram of differentially expressed exosomal miRNAs in OL-SCs-EXO and OL-EXO. **C**, **D** Differentially expressed miRNAs in cells and exosomes of OL and OL-SCs were analyzed by RT-qPCR. **E** The expression level of miR-1268a in OL cells with and without OL-SCs-EXO treatment was detected by PCR. **F** OL-SCs exosomes with cy5-labeled miR-1268a were add to OL cells, co-cultured with PKH26-labeled exosomes for 6 h, 9 h, 12 h, respectively. Fluorescence signals were detected confocal fluorescence microscope. Scale bar, 20 μm. Data were expressed as the mean $\pm$ SD, *p < 0.05, **p < 0.01, ***p < *0.001*

Since increasing evidence begins to elucidate the importance of exosomal miRNAs in cancer development and progression, we focused our mechanistic investigation on exosomal miRNAs. We conducted miRNA sequencing to profile differentially expressed miRNAs between OL-SCs and OL exosomes (Sect. “Materials and methods”). We identified differentially expressed miRNAs by using the following criteria: |log2(FC)|> 2 (FC: fold-change) and P < 0.001. Among the identified miRNAs, 7 were upregulated and 79 were downregulated in OL-SCs exosomes compared to OL exosomes (Fig. 3A, B). These findings show that exosomal miRNA differential expression pattern is a new feature of CSCs and non-CSCs.

Since we are searching for exosomal miRNAs secreted by OL-SCs that promote cancer metastasis, we focused our analysis, prioritization and validation on the 7 miRNAs that exhibited elevated expression in OL-SCs-EXO. We performed RT-qPCR verification of 7 up-regulated miRNAs in cells and exosomes of OL and OL-SCs. We found that compared to OL cells, miR-1268a is most abundantly expressed in OL-SCs cells, whether in cells or exosomes (Fig. 3C, D). These results suggested that miR-1268a may be involved in the malignant progression of melanoma. Moreover, literature search revealed that miR-1268a is a rather novel miRNA, especially in melanoma research. We thus prioritized miR-1268a for further mechanistic investigation.

We next confirmed that miR-1268a can be transferred from OL-SCs cells to OL cells by OL-SCs-EXO. The cellular expression level of miR-1268a was increased in OL cells upon treatment with OL-SCs-EXO (Fig. 3E), indicating that exosomes can transport miR-1268a into OL cells. In addition, we transfected OL-SCs cells with Cy5 labeled miR-1268a mimics (green). Exosomes were extracted and labelled with PKH26. PKH26-labeled OL-SCs-EXO were added to OL cells culture and confocal microscopy imaging was conducted to visualize the time course (6 h, 9 h and 12 h) of OL-SCs-EXO (PKH 26-labeled, red) uptake by OL cells and exosomal miR-1268a (Cy5-labeled, green) release within OL cells (Sect. “Materials and methods”). Shown in Fig. 3F, at 9 h, PKH26-labeled OL-SCs-EXO entered OL cells and accumulated mostly in the cytosol (Fig. 3F, 3rd lane, red). In addition, Cy5-labeled miR-1268a mimics were found inside OL cells (Fig. 3F, 2nd lane, green). Co-localization of Cy5-miR-1268a mimics with PKH26-OL-SCs-EXO (Fig. 3F, 4th lane, yellow) indicates that miR-1268a can be transferred from OL-SCs cells to OL cells via the route of exosomes. To better visualize the exosomal transferred miRNAs inside the cell body, we labelled the cell membrane with the cell membrane fluorescent dye DiO to more clearly observe the encapsulation of miR-1268a into OL cells upon exosomes transfer (Additional file 1: Figure S1A).

**Fig.4:**
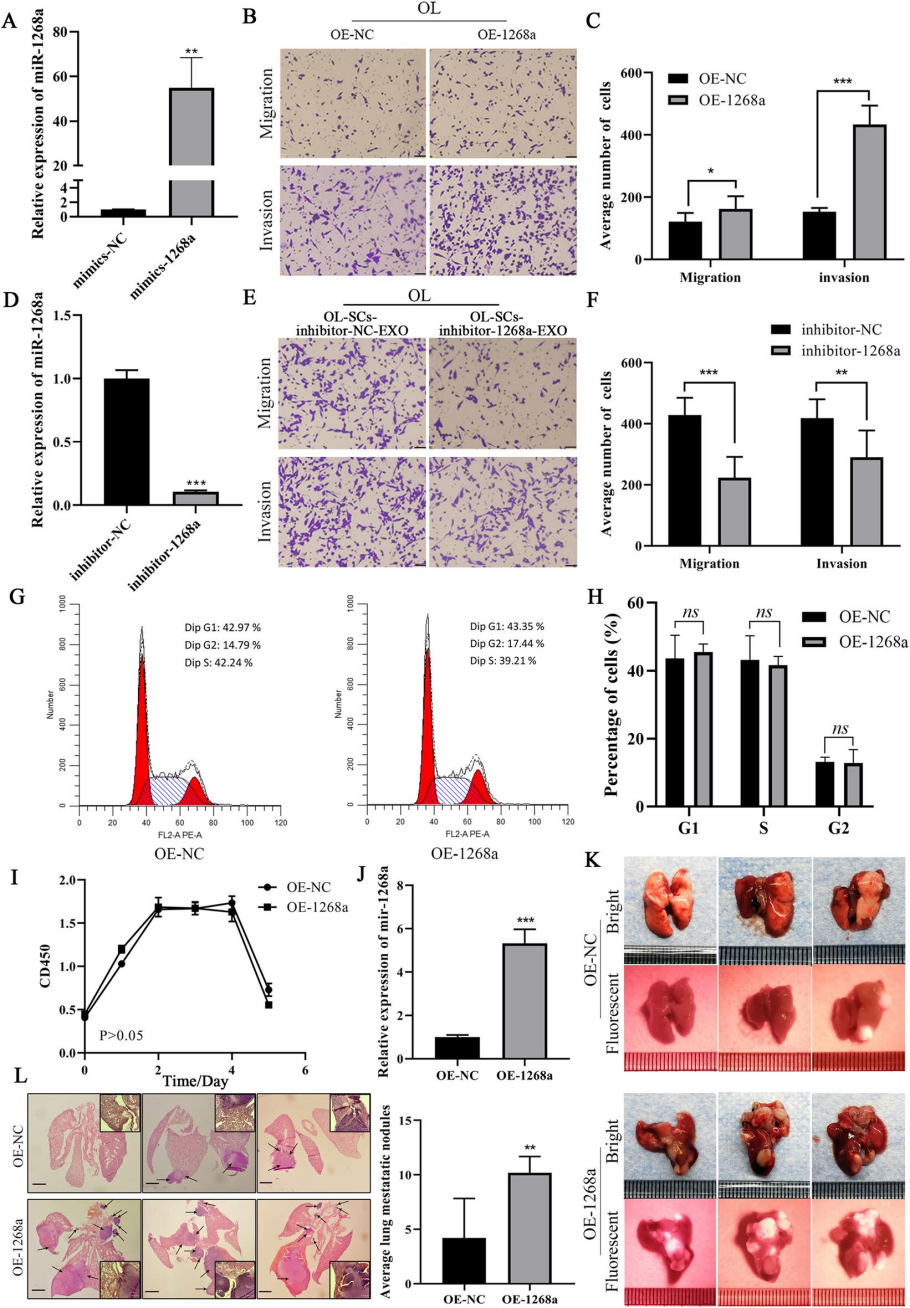
miR-1268a promotes the metastasis of melanoma in *vivo* and in *vitro*. **A** miR-1268a expression levels in OL cells transfected with miR-1268a mimics or NC mimics were detected by RT-qPCR. **B**, **C** Overexpression of miR-1268a promoted the migration and invasion ability of OL cells as revealed by Transwell assay. (Three replicate wells were set in each group, which were repeated 3 times.10 random fields from three replicate Transwell were counted. n = 3) Scale bar, 64 μm. **D** miR-1268a expression levels in OL-SCs cells transfected with miR-1268a inhibitor or NC inhibitor were detected by RT-qPCR. **E**, **F** OL-SCs transfected with miR-1268a inhibitor or NC inhibitor and then isolated exosomes added to OL cells, the invasion and migration capacity of OL cells treated with OL-SCs-inhibitor-NC-EXO、OL-SCs-inhibitor-1268a-EXO and PBS was assessed by Transwell assay. (Three replicate wells were set in each group, which were repeated 3 times.10 random fields from three replicate Transwell were counted. n = 3) Scale bar, 64 μm. **G** The effect of miR-1268a on changes in OL cell cycle distribution was determined by flow cytometry. **H** The proportion of cells in OE-miR-1268a-OL cells and OE-NC-OL cells at different phase (G1, S, G2), The experiment was repeated 3 times for each group. **I** The proliferation capacity of OL cells transfected with miR-1268a mimics or NC mimics were measured by CCK-8. **J** miR-1268a expression levels in OL cells stably infected with miR-1268a-overexpressing lentivirus and NC lentivirus were detected by RT-qPCR. **K** Whole-lung fluorescent and bright images of lung metastasis in NOD-SCID mice after tail intravenous injection of OL cells treated with miR-1268a overexpressing or NC lentiviruses, n = 6. Scale bars, 1 mm. **L** H&E-stained images of mouse whole lung sections after NOD-SCID mice were stably infected with miR-1268a-overexpressing lentivirus and NC lentivirus OL cells by tail vein injection, scale bar, 2 mm. Scale bar for magnified image, 530 μm. Data were expressed as the mean $\pm$ SD, *p < 0.05, **p < 0.01, ***p < *0.001*

To investigate the effect of miR-1268a on melanoma metastasis, we used mimics to overexpress miR-1268a in OL cells (Fig. 4A). miR-1268a overexpression (OE) augmented OL cells migration and invasion in vitro (Fig. 4B, C). To confirm that OL-SC-EXO miR-1268a regulates the migration and invasion of OL cells in vitro, we inhibited the expression of miR-1268a in OL-SCs cells using miR-1268a-inhibitor (Chemically modified inhibitor specifically targets miR-1268a, thus can compete with mature miR-1268a sequence for binding, weakening the gene silencing effect of endogenous miR-1268a) (Fig. 4D). Incubation of exosomes isolated from inhibitor-treated OL-SCs cells with OL cells resulted in a significant inhibition of OL cell migration and invasion compared to control mimics treated OL-SCs-EXO (Fig. 4E, F). To rule out that the enhancement of migratory and invasive abilities of OL cells by miR-1268a was caused by the differences in cell cycle or proliferative ability between OL cells transfected with mimics, we examined the cell cycle and proliferation of OL cells transfected with mimics-NC and mimics-miR-1268a using flow cytometry PI staining, CCK-8 proliferation assays and EdU-cell proliferation assay, respectively (Sect. “Materials and Methods”). Transfection of miR-1268a had no significant effect on the cell cycle progression (Fig. 4G, H, Additional file 1: Figure S1B-C). Furthermore, Transfection of miR-1268a also had no significant effect on OL cells proliferation (Fig. 4I, P > 0.05, Additional file 1: Figure S1D). Collectively, these results indicate that the observed changes in OL cells migration and invasion were not due to altered cell growth properties.

To study the effect of miR-1268a overexpression on OL cells metastasis in vivo, we generated OL cells stably overexpressing miR-1268a via lentiviral infection (Fig. 4J). $5 \times 10^{5}$ OE-NC-OL and OE-miR-1268a-OL cells were injected into NOD/SCID mice via the tail vein, respectively (Sect. “Materials and methods”). All mice were sacrificed at day 35 after tumor cell injection. Since OE-NC-OL and OE-miR-1268a-OL cells were RFP-labeled, macroscopic metastatic foci formed at the lung could be visualize under external fluorescence imaging using LUYOR-3415 Dual Fluorescent Protein Excitation Light Source and LUV-50A Glasses. As shown in Fig. 4K, injected OE-miR-1268a-OL cells produced significantly more macroscopic lung foci than the OE-NC-OL cells (n = 6). Subsequently, H&E analysis was performed on the largest cross-sectional lung sections of the two groups of mice. Statistical analysis showed that there were significantly more microscopic metastatic foci in the lungs of mice treated with OE-miR-1268a-OL cells (The black arrows show the formed metastases) (Fig. 4L). These observations show that miR-1268a can enhance metastatic colonization efficiency of OL cells that are oligometastatic. So far, we have demonstrated that miR-1268a, transferred from OL-SCs-derived exosomes to OL cells, can effectively augment the clonogenic colonization capability of OL cells to give arise to more extensive macroscopic metastases.

Collectively, these data indicated that OL-SCs cells secreted exosomal miR-1268a promotes the metastatic colonization ability of OL cells in vitro and in vivo.

### The pro-metastatic activity of exosomal miR-1268a is achieved through inhibition of autophagy

**Fig.5:**
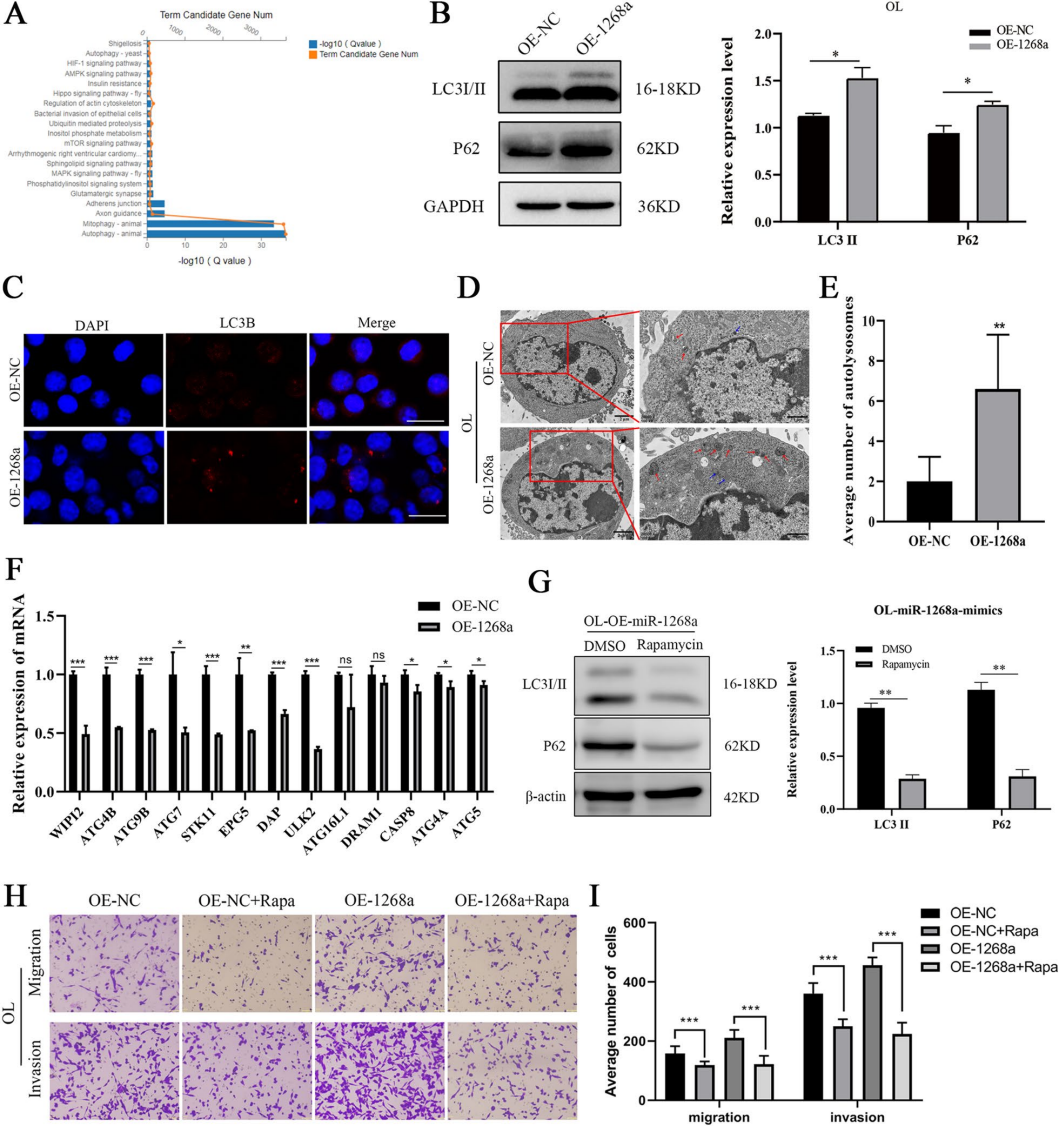
miR-1268a enhance invasion and migration capacity by inhibiting autophagy pathway. **A** BGI Database to determine miR-1268a predicted target genes and to analyze pathway enrichment of the predicted target genes. **B** LC3I/II and P62 protein expression in OL cells transfected with miR-1268a mimics or NC mimics were analyzed by WB, the right side is the grayscale analysis chart of WB (the ordinate is the target protein/GAPDH). **C** Fluorescence image of positive-LC3 fusion protein in OL cells transfected with miR-1268a mimics or NC mimics. Scale bar, 20 μm. **D**, **E** Transmission electron microscopy of autolysosomes in OL cells transfected with miR-1268a mimics or NC mimics (Primary lysosomes are marked with blue arrows and autolysosomes are marked with red arrows). Scale bar, 2 μm,1 μm. **F** Expression of some autophagy-related genes in OL cells transfected with miR-1268a mimics or NC mimics were detected by RT-qPCR. **G** LC3I/II and P62 protein expression in OE-miR-1268a-OL cells treated with DMSO and Rapamycin (10μΜ) were analyzed by WB, the right side is the grayscale analysis chart of WB (the ordinate is the target protein/β-actin). **H**, **I** The migration and invasion ability of OE-miR-1268a-OL and OE-NC-OL cells treated with autophagy activator rapamycin by Transwell analysis. (Three replicate wells were set in each group, which were repeated 3 times.10 random fields from three replicate Transwell were counted. n = 3) Scale bar, 64 μm. Data were expressed as the mean $\pm$ SD, *p < 0.05, **p < 0.01, ***p < *0.001*

To explore the molecular mechanisms that mediate the oncogenic activities of exosomal miR-1268a, we used Target Scan Human (http://www.targetscan.org/) and BGI Database to determine its predicted gene targets and to analyze pathway enrichment. We found that most of the predicted target genes of miR-1268a were enriched in the “autophagy pathway”, suggesting that miR-1268a may regulate autophagy (Fig. 5A). To validate whether miR-1268a may regulate autophagy, three classical autophagy research methods were used to characterize the autophagy status when miR-1268a expression was altered by transfection of mimics: (1) protein levels of LC3 and P62 were detected by Western blotting (WB), (2) mRFP-LC3B punta was visualized by immunofluorescence (IF), and (3) the accumulation of autolysosome in the cytoplasm was analyzed by transmission electron microscopy (TEM). We found that stable overexpression of miR-1268a inhibited autophagy in OL cells, supported by: accumulation of LC3/P62 (Fig. 5B), increased mRFP-LC3B punta (Fig. 5C) and accumulation of autolysosomes (Fig. 5D, E). Subsequently, we measured the expression levels of a panel of autophagy-related and predicted miR-1268a target genes by RT-qPCR. Most of them showed decreased expression in OL cells that miR-1268a was overexpressed (Fig. 5F). These results show that miR-1268a can effectively inhibit the autophagy pathway by targeting multiple targets of the pathway in OL cells.

Our prior study has shown that activation of autophagy in OL cells hinders OL cell invasion both in vitro and in vivo [39]. Thus, inhibition of autophagy upon miR-1268a overexpression in OL cells should result in augmented migration and invasion. To confirm that autophagy pathway mediates the pro-metastatic activity of miR-1268a in OL cells, the autophagic activity was stimulated by Rapamycin (Rapa) treatment. As shown in Fig. 5G, the autophagy inhibited by overexpression of miR-1268a was reversed by treating OE-miR-1268a-OL cells with Rapa. Activation of autophagy by Rapa in OE-miR-1268a-OL cells prevented the increase of cell migration and invasion in vitro caused by miR-1268a overexpression (Fig. 5H, I). These observations indicate that OL-SCs exosomal miR-1268a, upon taking up by OL cells, can promote metastatic colonization through targeting the autophagy pathways.

Collectively, these results indicated that melanoma stem cells can enhance the metastatic capability of low metastatic non-CSCs OL cells by transferring of their exosomal miR-1268a to the OL cells which in turn targets the autophagy pathway.

## Discussion

Metastasis is the main cause of treatment failure and death in cancer. Although melanoma accounts for only 5% of all skin cancers, it accounts for 75% skin cancer-related deaths [3]. Patients with metastatic melanoma have very limited treatment options [1]. More effective clinical management of melanoma requires improved understanding of the molecular mechanisms underlying metastatic progression of this disease. The high mortality rate of metastatic melanoma is related to the efficient metastatic colonization at the involved organs. Proliferative colonization is the rate-limiting step of metastasis [40–42], that requires the formation of a new microenvironment.

The role of exosomes in the establishment of metastatic microenvironment (MME) has gained increasing attention in cancer research. The exosomal crosstalk involves the transfer of “biologically active material” including miRNA, mRNA, lncRNA and proteins between the donor cells and the neighboring recipient cells, thus is capable of altering the biological properties of the recipient cells [10, 43]. Published studies in recent years have shown that exosomes can mediate the interactions between melanoma cancer cells and stromal cells, as well as between cancer cells and normal melanocytes, which may play an important role in melanoma oncogenesis and progression [44–49].

CSCs are a subset of heterogeneous cells with self -renewal capability. CSCs may play an important role in melanoma metastasis and the development of drug resistance [50]. Exosomes secreted by CSCs are shown to be regulators of tumor microenvironment, capable of changing the fate of non-CSCs target cells [7]. CSCs-derived exosomal miRNAs can promote metastatic progression in renal cancer [25], lung cancer [51] and in glioma [52]. However, the effect of exosomes secreted by CSCs on melanoma metastasis has not been reported.

This study has made the following novel findings that have elucidated a new CSCs-exosome-based mechanism that underlies melanoma progression

### First, CSCs can enhance the metastatic colonization capability of non-CSCs melanoma cells through exosomal crosstalk

CSCs can communicate with the tumor microenvironment by releasing a variety of growth factors, interleukins, cytokines and extracellular vesicles, and participate in tumor metastasis, drug resistance and other processes [7]. In colon cancer, IL-4 secreted by CSCs is required for the maintenance of stemness and for inhibiting apoptosis [53]. TGF-ß expressed and released by CSCs affects tumor cell invasion and metastasis[54]. The role of miRNAs in mediating the interactions between CSCs and tumor microenvironment has been reported [7, 15].

Previous studies on the role of exosomes in metastasis have been largely focused on cancer cell-derived exosomes in modifying the functions of stromal cells in the tumor microenvironment [55]. Whether the extravasated CSCs at the distant organ can modify the metastatic properties of the neighboring non-CSCs, at the stage of metastatic colonization, the rate-limiting stage for metastasis, has not been demonstrated prior to this study.

The lack of clinically relevant metastatic melanoma cellular and in vivo models as well as stable CSCs models has limited the in-depth mechanistic investigation. In this study, we employed a pair of M14-derived paired cell lines, OL and OL-SCs, which we generated and characterized in previous studies [34, 35]. M14-OL cells formed a limited number of metastatic foci (defined as oligometastatic-OL) on mouse lungs after tail vein injection in vivo. OL-SCs represents the CSCs component of the OL as we characterized. Exosomal profiling provides a new molecular feature that can differentiate CSCs and non-CSCs. The availability of this paired cell lines allows us to show that melanoma CSCs can increase metastatic efficiency by augmenting the metastatic colonization capability of non-CSCs through the mechanism of exosomal transfer (Fig. 3).

### Second, exosomal miR-1268a from highly metastatic melanoma CSCs augments the migration and invasion of low metastatic non-CSCs cancer cells

The involvement of miR-1268a in angiogenesis, embryo and cell differentiation [56], drug resistance [57] and cancer progression [58] has been reported. However, mechanistic understanding of miR-1268a in metastasis is lacking, and its involvement in melanoma is unknown. Further, the function of miR-1268a in exosomal crosstalk has not been demonstrated prior to this study. In the present study, we have demonstrated that miR-1268a, transferred from OL-SCs-derived exosomes to OL cells, can effectively augment the clonogenic colonization capability of OL cells in vitro and in vivo (Figs. 3, 4).

In addition to miR-1268a, five miRNAs that are significantly increased in OL-SCs cells (miR-93-3p, miR-4281, miR-592, miR-1246 and miR-4535). These miRNAs act as oncogenes in most cancer types, promoting tumor cell proliferation, clonality, migration, invasion, and metastasis [59–61]. Their high expression in OL-SCs cells may also contribute to melanoma progression. Among them, the pro-metastatic activity of miR-4535 is also achieved by inhibiting autophagy [62]. Therefore, it is quite possible that miRNAs exhibited significantly high expression in CSCs may act coordinatively or synergistically to promote tumor metastasis by targeting the autophagy pathway or other biological processes. Verification of this hypothesis requires follow-up studies. In addition, the above-mentioned miRNAs have shown the possibility of being targets for tumor therapy in several studies. Similarly, miR-1268a has also been reported to be a marker of poor prognosis in liver cancer [58]. Whether miR-1268a is a potential intervention target for melanoma treatment requires validation with clinical samples.

Although exosomal miR-1268a has changed the metastasis of OL cells in vitro and in vivo, whether CSCs can modify the stemness features of non-CSCs via exosomal interactions is an interesting speculation. To test this hypothesis, we verified the effect of OL-SCs-EXO on the stemness of OL cells by 96-well plate spheroidization experiment, 6-well plate spheroidization experiment and stem cell markers analysis. As shown in Additional file 1: Figure S1E-F, the pro-metastatic effect of OL-SCs-EXO is unlikely achieved by affecting the stemness of OL cells.

### Third, the pro-metastatic activity of CSCs exosomal miR-1268a is achieved through inhibition of the autophagy pathway in non-CSCs cells

Although there are few studies showing the oncogenic activity of miR-1268a, mechanistic understanding in lacking. The function of exosomal miR-1268a has not been reported. The pro-metastatic function of exosomal miR-1268a that we have observed is consistent with the literature reports [57, 58].

To investigate the molecular mechanisms underlying the pro-metastatic activity of exosomal miR-1268a, we performed bioinformatics analysis of predicted gene targets of miR-1268a. Pathway enrichment analysis has identified “autophagy pathway” that is most pronouncedly associated with differential miR-1268a expression (Fig. 5A). Biological validation confirmed the inverse relationship between a panel of autophagy-associated predicted gene targets of miR-1268a and miR-1268a expression (Fig. 5F). These findings show that miR-1268a may regulate autophagy in OL cells by simultaneously targeting multiple genes that participate in the autophagic process, i.e., by pathway targeting. This mode of pathway targeting will be more efficient than that of targeting a single gene of the same pathway. Thus, pathway targeting is a mechanism of exosomal miRNA that can effectively modify the metastatic phenotype of the target cells.

The role of autophagy in tumor metastasis is complex. Both the anti- metastatic and pro-metastatic roles of autophagy have been reported and appear to be context and stage-dependent [63]. During the clonogenic colonization stage of metastasis, on the one hand, newly extravasated circulating tumor cells reach the pre-metastatic niche, and autophagy can keep these tumor cells in the dormant stage, preventing proliferation and colonization [64, 65]. On the other hand, once micro-metastases are formed, autophagy can promote metastasis by helping tumor cells adapt to the stressful foreign microenvironment [66].

miRNAs are an important class of regulators of autophagy. miRNAs can regulate upstream signaling pathways of autophagy, such as PI3K-AKT-mTOR, TP53-mTOR, and Ca^2+^-AMPK-mTOR, or can directly target Autophagy-related genes [67, 68]. Our previous research shows that activation of autophagy through the "SEC23A-S1008-BECLIN1-autophagy axis" [39] inhibits melanoma metastasis at the step of metastatic colonization. In this study, we show that miR-1268a promotes metastasis by targeting autophagy pathway. The specific mechanism underlying miR-1268a regulation of autophagy requires further clarification and merits future investigations.

## Conclusion

we show for the first time that exosomal crosstalk between the melanoma stem cells and the low-metastatic non-CSCs melanoma cells is a new mechanism that underlies melanoma metastasis and heterogeneity. Highly metastatic melanoma CSCs (OL-SCs) can transfer their “metastatic capacity” to low-metastatic melanoma cells (OL). OL-SCs achieve this goal by utilizing its exosomes to deliver functional miRNAs, such as miR-1268a, to the targeted OL cells which in turn inactivates autophagy pathway and augments metastatic colonization. The clinical relevance of this finding will be addressed in future studies.

## Materials and methods

### Cell lines and cell culture

M14 cells were kindly provided by Dr. Robert Hoffman (University of California San Diego). As described in our previous studies, M14-OL, a M14 derivative cell line was established via three-rounds of in vivo passage, isolation and purification. The established OL cells, upon tail-vein injection, form limited number of metastatic foci (defined as oligometastatic–OL) on the mouse lungs [34]. Melanoma Stem Cells (OL-SCs) were isolated and purified from OL cells using the method we previously described [35, 69]. OL cells were maintained in DMEM high glucose supplemented (Gibico, USA) with 10% fetal bovine serum (FBS) (Gibico, USA) and 1% penicillin–streptomycin (Hyclone, USA). OL-SCs were cultured in DMEM/F12-based normal stem-cell media (Hyclone, USA) supplemented with 2% B27 (Gibico, USA) and 1% penicillin–streptomycin (Hyclone, USA).

### Exosome isolation and identification

OL and OL-SCs cells were cultured in petri dishes (diameter:10 cm, Thermo Fisher Scientifc, USA) containing 12–15 ml of cell culture medium, and the culture medium of 10 dishes was collected each time for exosome extraction. Exosomes were obtained by differential ultracentrifugation. Cells were harvested when OL and OL-SCs cultures reached 90% confluency. After centrifugation at 800 g for 5 min, the supernatants were harvested and centrifuged at 2000 g at 4 °C for 10 min. The collected supernatant was rapidly concentrated using a centrifugal concentrator with a pore size of 100 kDa MWCO (Millipore, USA). For differential ultracentrifugation, the supernatants collected from low-speed centrifugation were centrifuged at 10000 g at 4 °C for 10 min and then passed through a 0.22 μm filter (EMD Millipore, USA). Thereafter, the supernatants were centrifuged at 100000 g at 4 °C for additional 70 min, the exosome pellets were washed with 2 ml of Phosphate-buffered saline (PBS, Hyclone, USA) and resuspended in 35 ml of PBS, and the sample was centrifuged again at 100,000 g for 70 min. The resultant pellets were resuspended in 200-300 μl PBS and stored at -80 °C for further analysis.

Protein content of the exosomes isolated were determined by using a micro-BCA Protein Assay Kit (CWBIO, China). Morphology of exosomes were analyzed using Transmission electron microscopy (TEM, JEM-1400PLUS, JEOL). The size distribution and concentration of exosomes were determined by Nanoparticle Tracking Analysis (NTA). Western blot(WB)was used to characterize the expression of exosomal markers.

### Western blot(WB)

Total cell proteins were extracted by RIPA (Solarbio, China). Exosomal proteins were extracted by repeated freeze–thaw lysis at -80℃. Cellular and exosomal protein concentrations were measured by BCA protein assay kit (CWBIO, China) and micro-BCA protein assay kit, respectively. Each protein sample (20 μg) was separated by electrophoresis with 12% polyacrylamide gels and transferred to polyvinylidene fluoride (PVDF) membranes (Bio-Rad, USA). Subsequently, PVDF membranes were blocked, incubated with antibodies and washed. Primary antibodies of Alix, CD63, CD81, Calnexin, RAB27a, and the loading controls (GAPDH and β-Actin) were purchased from Proteintech Group, USA. LC3, P62 and TSG101 antibody was purchased from Abcam, Britain. The secondary Antibodies: anti-Rabit and anti-Mouse was purchased from Proteintech Group.

### Exosome treatment

Exosome treatment was used in migration and invasion Transwell assays and PCR experiments. Protein concentration of the prepared exosomes were determined by the micro-BCA Protein Assay Kit (CWBIO, China). For Transwell analysis, when treated with exosomes secreted by OL-SCs cells (OL-SCs-EXO), OL cells were seeded in the upper chamber of the chamber and co-cultured with OL-SCs-EXO (40 μg) for 24 h. When treated with exosomes derived from OL-SCs with knockdown of the RAB27a gene (OL-SCs-shRAB27a-EXO), the culture medium of the same number of shRAB27a-OL-SCs cells and shNC-OL-SCs cells was collected for the extraction of exosomes. During the Transwell migration and invasion assay, equal volumes of OL-SCs-shRAB27a-EXO, OL-SCs-shNC-EXO, and PBS were added to the upper chamber to co-culture with OL cells for 24 h. When OL cells were treated with exosomes secreted by OL-SCs cells transfected with NC-inhibitor and miR-1268a-inhibitor (OL-SCs-NC-inhibitor-EXO, OL-SCs-1268a-inhibitor-EXO). OL cells were seeded in the upper chamber of the chamber, and OL-SCs-NC-inhibitor-EXO and OL-SCs-1268a-inhibitor-EXO of the same quality (40 μg) were added to co-culture for 24 h. For PCR experiments, OL cells were seeded in six-well plates and cultured for 48 h, and OL-SCs-EXO (60 μg) and an equal volume of PBS were added to continue to culture for 24 h, and the cells were collected.

### Transwell migration and invasion assays

Cell invasion and migration was performed in triplicate using 24-well Transwell plate (8 μm pore size, BD Falcon, USA), and were used to perform cell invasion assay with Matrigel (1:10 dilution in serum free medium, BD Biosciences, USA) and migration assay without Matrigel. For the migration assay, 30,000 cells were placed in the upper chamber supplemented with 300 μl serum-free DMEM medium and 800 μl DMEM medium containing 20% FBS in the lower chamber. After culturing for 24 h, the chamber was taken out and washed with PBS to remove floating cells. Subsequently, the cells on the chamber membrane were fixed with ice methanol for 20 min, washed twice with PBS to remove residual methanol, and stained with crystal violet for 5–10 min. Next, the stained chamber was washed twice with PBS to remove the floating color, and the cells on the membrane surface of the upper chamber were removed by gently wiping with a cotton swab. Finally, the dried chamber was photographed under a microscope and 10 random fields from three replicate wells were counted by the software image J. The basic steps of the invasion assay are similar to that of the migration assay. The difference is that in the invasion assay, the chamber needs to be coated with Matrigel 24 h in advance, and 50,000 cells were added. Each experiment was repeated 3 times.

### Lentivirus and oligonucleotide transfection

The lentivirus particles of NC, miR-1268a overexpression (OE) and RAB27a silencing (sh-RAB27a) plasmids were purchased from Shanghai GenePhaema Company (China). miR-1268a mimics, miR-1268a inhibitor and negative control were chemically synthesized by GenePhaema (Shanghai, China). Cells were infected with lentivirus or transfected with siRNA and mimics by Lipofectamine 2000 (Thermo Fisher Scientifc, USA) according to the manufacturer's instructions, respectively. After 72 h of infection and 48 h of transfection, the overexpression and silencing of target genes or miRNAs were analyzed by PCR to verify the infection and transfection efficiency.

### Real-time quantitative polymerase chain reaction (RT-qPCR)

Total RNA from cells and exosomes was extracted using Trizol (Invitrogen, USA) and miRNeasy Serum/Plasma Kit (QIAGEN, Germany), respectively. Reverse transcription was performed using PrimeScript RT Master Mix (Takara, Japan) and Mir-X™ miRNA First Strand Synthesis Kit (Takara, Japan). RT-qPCR was conducted using SYBR Green Real-time PCR Master Mix Kit (Takara, Japan) according to the manufacturer’s instructions and the following PCR condition: in a 10 μl reaction volume, initial pre-incubation at 95 ℃ for 30 s, followed by 39 cycles at 95 ℃ for 5 s and 60 ℃ for 30 s.The relative expression level of miRNAs was calculated through normalization to U6 internal controls, and mRNAs were normalized with GAPDH. The primers used for PCR are shown in Table 1.

**Table 1:** PCR primer sequence

Gene name	Forward primer (5′‐3′)	Reverse primer (5′‐3′)
WIPI2	AGGAGAGGAGTGAAGAGGTGTGTG	GTTGCTGGATGCGGAGAGGAAC
ATG4B	GCGGCTGCACTTCCTACTGATTC	CCAGGCGGAGAGGGATGAGAAG
ATG9B	CTTCATCAACAGCAGCAGCAAGAAC	TAGAAGCAGGACTGGAGCCATCAC
ATG7	CCCAGAAGAAGTTGAACGAGTACCG	GCAGACCAGCAGAGTCACCATTG
STK11	GCCCACCACTCTCTGACCTACTC	CTGTGCTGCCTAATCTGTCGGATG
EPG5	CCGTTCTGGTGTCCCTTCAAGTTC	GTCGTTCTTCACCGTCTGCTCAC
DAP	GGAAAGCACCAGCCCACCTA	CGTGTTTGTCCATGGAGGCG
ULK2	AGACACCTTACGCCATCTGAACATG	TCTGGTCCACAACTACACTCTCCTG
ATG16L1	CAAGCCGAATCTGGACTGTGGATG	CAGGAACTTGGCAGAGAGGACTTTC
DRAM1	GTCGTCCGCAGCCTTCATCATC	TCTCTGGAGGAGTTGTTCCTGTGTC
CASP8	GGATGTTGGAGGAAGGCAATCTGTC	GGGAGAAATCTGGGCATTGTCTGG
ATG4A	TGGACCAAACACAGTTGCACAGG	CAGCAGCACCCACAGGAAGAAC
ATG5	ATGCGGTTGAGGCTCACTTTA	TTTCCGGTTGATGGCCCAAA
RAB27a	TCTGGTGTAGGGAAGACCAGT	TACGAAACCTCTCCTGCCCT
GAPDH	ACCCTTAAGAGGGATGCTGC	CCCAATACGGCCAAATCCGT
U6	CTCGCTTCGGCAGCACA	AACGCTTCACGAATTTGCGT
miR-4535	GTGGACCTGGCTGGGAC	
miR-93-3p	ACTGCTGAGCTAGCACTTCCC	
miR-1246	CGCGAATGGATTTTTTGGAGCAGG	
miR-1268a	TATATATACGGGCGTGGTGGTGGG	
miR-592	CGCGTTGTGTCAATATGCGATGATGT	
miR-4281	TATATATATAGGGTCCCGGGGAGGGG	
miR-3529-3p	CGCGCGAACAACAAAATCACTAGTCTT	

### miRNA Sequencing and analysis

Triplicates of OL-SCs cell exosomes and OL cell exosomes extracted by ultracentrifugation were sent to BGI for miRNA sequencing and data analysis (https://biosys.bgi.com/). For miRNA sequencing, total RNA in OL and OL-SCs exosomes was extracted. Total RNA was identified and quantified using NanoDrop and an Agilent 2100 Bioanalyzer (Thermo Fisher Scientific, MA, USA). Library was prepared with 1 μg total RNA for each sample. Total RNA was purified by electrophoretic separation on a 15% urea denaturing polyacrylamide gel electrophoresis (PAGE) gel and small RNA regions corresponding to the 18–30 nt bands in the marker lane (14–30 ssRNA Ladder Marker, TAKARA, Japan) were excised and recovered. Then the 18–30 nt small RNAs were ligated to adenylated 3’ adapters annealed to unique molecular identifiers (UMI), followed by the ligation of 5’adapters. The adapter-ligated small RNAs were subsequently transcribed into cDNA by SuperScript II Reverse Transcriptase (Invitrogen, USA), followed by several rounds of PCR amplification with PCR Primer Cocktail and PCR Mix to enrich for cDNA fragments. The PCR products were seperated by agarose gel electrophoresis. Target fragments (110 ~ 130 bp) were cut and purified by QIAquick Gel Extraction Kit (QIAGEN, Valencia, CA). Fragment size distribution was examined using an Agilent 2100 Bioanalyzer, and quantitative detection of the library was performed using quantitative real-time PCR (TaqMan probes). The final ligation PCR products were sequenced using the BGISEQ-500 platform (BGI-Shenzhen, China). For data analysis, the raw sequencing data is pre-processed by quality assessment and data filtering, and then the obtained sequences are aligned and annotated with the miRNA database to obtain known miRNAs. For the sequences that were not aligned and annotated in the sequencing results, they were compared with the whole genome sequence of the species, and the sequence was preliminarily judged to be an unknown new miRNA. Next, the samples (OL-SCs-EXO, n = 3; OL-EXO, n = 3) were used for comparative analysis of miRNA expression using the DEGseq toolkit. The filter condition is: |log2(FC)|> 2 (FC: fold-change), P < 0.001.

### Detection of Cy5-labelled miR-1268a transfer

OL-SCs was transfected with Cy5 labeled miR-1268a (GenePhaema, China), and extracted its exosomes by the above method of separation and purification of exosomes. Then OL-SCs exosomes were stained with PKH26 (Sigma, USA) according to the manufacturer’s instructions. After OL was cultured on 24-well plate cell slides for 24 h and respectively co-cultured with stained exosomes (40 μg) for 6 h,9 h,12 h. Next, the cells were fixed with ice ethanol, washed with PBS, and the nucleus stained with DAPI. Images were acquired with a confocal fluorescence microscope.

### Cell cycle

Cells were subjected to cell cycle analysis by flow cytometry. OL cells were transfected with miR-1268a-mimics and NC-mimics, respectively. 48 h after transfection, cells were harvested by centrifugation at 800 rpm/min for 5 min and residual medium was washed with PBS. Next, cells were fixed with 70% ethanol overnight at − 20 °C, washed with PBS, resuspended in 500 μl PBS, and then cells were stained with PI/RNase staining solution (1.25 mg/ml PI and 2 mg/ml RNase A) in the dark at 37 °C for 30 min. After incubation, cells were analyzed for DNA content using a flow cytometer (FACS Vantage SE, BD, America) and the results were analyzed using ModFit LT software. Triplicates were submitted for flow cytometry analysis.

### CCK-8 Cell proliferation assay

Cell proliferation analysis was performed using the Cell Counting Kit-8(CCK-8, Beyotime, China). 2000 cells were seeded in each well of a 96-well plate, and five replicate wells were set in each group. After the cells adhered, 10 μl of CCK-8 reagent was added to each well and incubated at 37 °C for 2 h. The absorbance at 450 nm of each well was detected by a microplate reader. Cell proliferation activity was measured for 5 consecutive days and cell proliferation curve was plotted.

### EdU analysis

Cell proliferation was detected using the BeyoClick™ EdU-488 Cell Proliferation Assay Kit (Beyotime, China). After OL cells were transfected with mimics for 24 h, pre-warmed EdU solution (10uM) was added and incubated for 2 h. Then the cells were collected and fixed with fixative (immunostaining fixative P0098) at room temperature for 15 min. The fixed cells were washed 3–5 times with washing solution (immunostaining washing solution P0106), treated with permeabilizing solution (immunostaining strong permeabilizing solution P0097) and incubated for 10–15 min. Then cells were washed once again. The Click reaction solution (Click Reaction Buffer, CuSO4, Azide 488, Click Additive Solution) was prepared according to the kit instructions. Finally, 0.5 ml of Click reaction solution was added to each well, and incubated at room temperature for 30 min in the dark. The Click reaction solution was removed by suction. After three washes, cells were observed under a fluorescence microscope and the fluorescence was detected by a multi-function microplate reader.

### Animal experiments

All NOD-SCID mice used by experiments were housed in the Animal Experiment Center of Chongqing Medical University. Animal experiments were performed in accordance with approved protocols and in accordance with the Institutional Animal Welfare Guidelines of Chongqing Medical University, and all NOD-SCID mice used in the study were euthanized by cervical dislocation.$5 \times 10^{5}$ OL cells of stably transfected with the miR-1268a overexpression vector (OL-miR-1268a-OE) or the control lentiviral vector (OL-OE-NC), prepared in 100 μl PBS were transplanted into each male NOD/SCID mouse via tail intravenous injection, respectively (OE-1268a group: n = 6; OE-NC group: n = 6). Mice were weighted every 2 days and dissected when the weight loss lasted for one week. Lung-derived metastatic nodules were examined and quantified by whole-lung bright, fluorescent and H&E images.

### Autophagy flux analysis of LC3B puncta

Autophagy flux analysis of LC3B puncta was conducted as we previously reported [34]. According to the manufacturer's instruction, the cell was infected with adenoviral-expressing mCherry-GFP-LC3B fusion protein (Beyotime, China) and the accumulation of autophagosomes is quantified by counting mRFP-LC3 puncta around the cells.

### Transmission electron microscopy (TEM) analysis of autolysosome

Cells were pelleted by centrifugation at 1200 rpm/min for 10 min. Cells were fixed with 2.5% glutaraldehyde for 24 h and 1% osmium tetroxide for 1 h and rinsed with 0.1 M phosphoric acid rinse. Subsequently, cells were dehydrated sequentially with graded ethanol (50%, 70%, 90%) and 90% acetone at 4 °C, and 3 times with 100% acetone at room temperature. Next, the cells are embedded, solidified and sectioned. Sections were stained with 3% uranyl acetate-lead citrate and photographed with a JEM-1400Plus transmission electron microscope (JEOL Ltd, Japan) to observe autolysosome.

### Statistical analysis

All statistical analysis in this study were performed with GraphPad Prism 8.0 software and presented as mean ± SD. Data were analyzed for normal distribution by Normality and Log Normality tests or Normality of residuals (Q-Q Plots, Anderson-Daring, D’Agostino & Pearson test, Shapiro–Wilk test, Kolmogorov–Smirnov test). Two groups were compared using Student's t test, and multiple groups were analyzed using one-way analysis of variance (ANOVA). All experiments were repeated at least three times. P < 0.05, P < 0.01 and P < 0.001 were considered statistically significant and were marked with an asterisk, double asterisks and triple asterisks, respectively.

## Supplementary Information

**Additional file 1: Fig. S1.** A The process of miR-1268a derived from OL-SCs cells entering OL cells through exosomes (Cy5 marks miR-1268a, PKH26 marks exosomes, and DiO marks cell membrane). Scale bar, 10 nm. B, C Graphs of the results of the remaining two replicate experiments in the cell cycle. D The proliferation ability of OL cells transfected with mir-1268a mimics and NC mimics was detected by EdU analysis, *p* > *0.05*. E The spheroidization ability of OL cells after OL-SCs-EXO treatment was detected by the six-well plate spheroidization assay. F Expression of stemness markers in OL cells after OL-SCs-EXO treatment by RT-qPCR, *p* > *0.05*.

## Data Availability

The data used to support the findings of this study are available from every author upon request.
