# Supplementary figures and images for: Melanoma stem cells promote metastasis via exosomal miR-1268a inactivation of autophagy
Source: Biol Res. 2022 Oct 1;55:29. doi: 10.1186/s40659-022-00397-z (PMC9526915; doi:10.1186/s40659-022-00397-z)

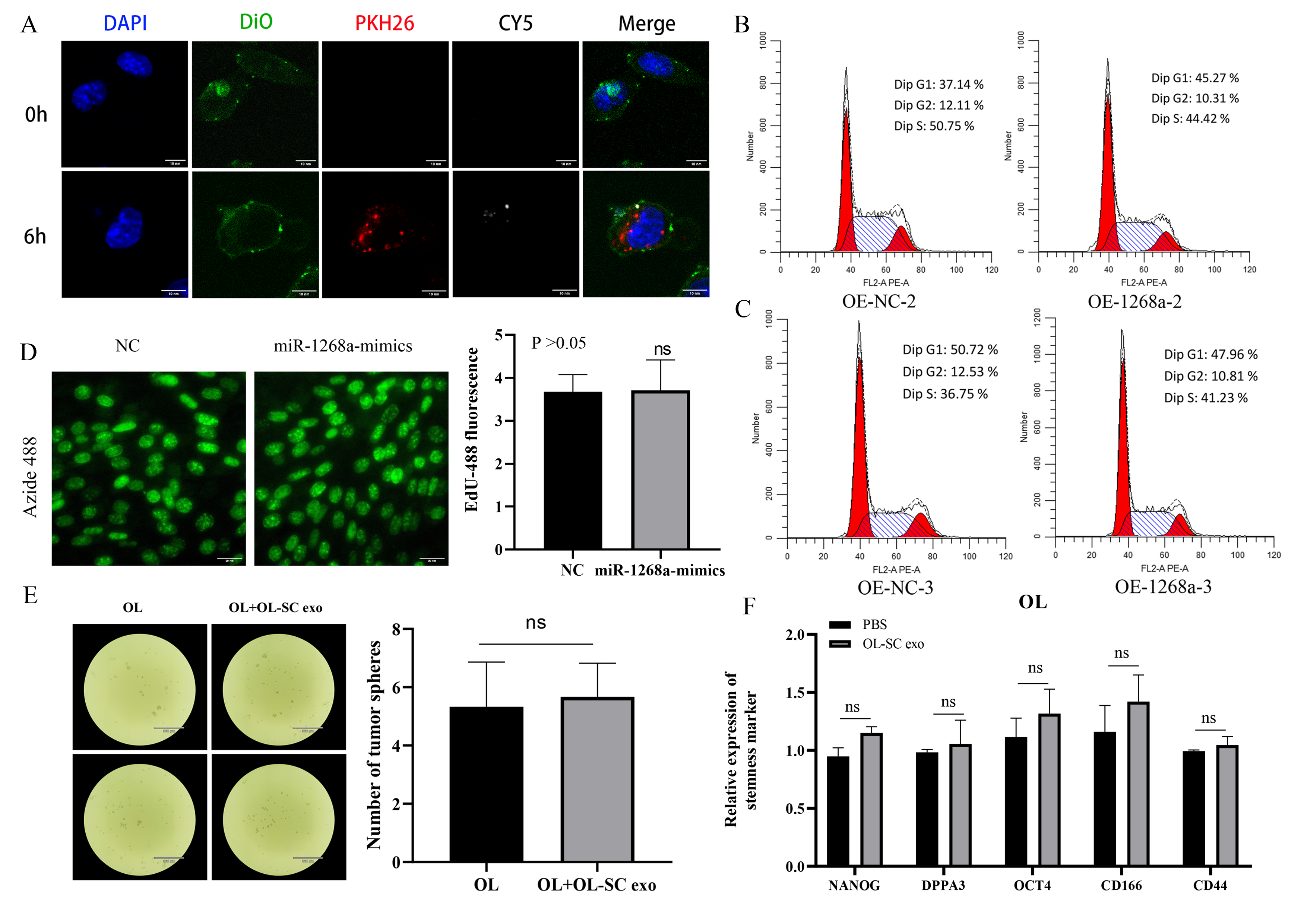

Supplement: Supplementary file 1 — Additional file 1: Fig. S1. A The process of miR-1268a derived from OL-SCs cells entering OL cells through exosomes (Cy5 marks miR-1268a, PKH26 marks exosomes, and DiO marks cell membrane). Scale bar, 10 nm. B, C Graphs of the results of the remaining two replicate experiments in the cell cycle. D The proliferation ability of OL cells transfected with mir-1268a mimics and NC mimics was detected by EdU analysis, p > 0.05. E The spheroidization ability of OL cells after OL-SCs-EXO treatment was detected by the six-well plate spheroidization assay. F Expression of stemness markers in OL cells after OL-SCs-EXO treatment by RT-qPCR, p > 0.05. [file 40659_2022_397_MOESM1_ESM.tif]
